# Future directions in meditation research: Recommendations for expanding the field of contemplative science

**DOI:** 10.1371/journal.pone.0205740

**Published:** 2018-11-07

**Authors:** Cassandra Vieten, Helané Wahbeh, B. Rael Cahn, Katherine MacLean, Mica Estrada, Paul Mills, Michael Murphy, Shauna Shapiro, Dean Radin, Zoran Josipovic, David E. Presti, Michael Sapiro, Jan Chozen Bays, Peter Russell, David Vago, Fred Travis, Roger Walsh, Arnaud Delorme

**Affiliations:** 1 Research Department, Institute of Noetic Sciences, Petaluma, California, United States of America; 2 Department of Neurology, Oregon Health & Science University, Portland, Oregon, United States of America; 3 Department of Psychiatry, University of Southern California, Los Angeles, California, United States of America; 4 Center for Optimal Living, New York, New York, United States of America; 5 Institute for Health and Aging, School of Nursing, University of California, San Francisco, San Francisco, California, United States of America; 6 Department of Family Medicine and Public Health, University of California, San Diego, San Diego, California, United States of America; 7 Center for Theory and Research, Esalen Institute, Big Sur, California, United States of America; 8 Department of Counseling Psychology, Santa Clara University, Santa Clara, California, United States of America; 9 Department of Psychology, New York University and Nonduality Institute, New York, New York, United States of America; 10 Department of Molecular and Cell Biology, University of California, Berkeley, California, United States of America; 11 Randall Children’s Hospital, Portland, Oregon, United States of America; 12 Osher Center for Integrative Medicine, Vanderbilt University Medical Center, Nashville, Tennessee, United States of America; 13 Center for Brain, Consciousness and Cognition, Maharishi University of Management, Fairfield, Iowa, United States of America; 14 School of Medicine, University of California, Irvine, California, United States of America; Universita degli Studi di Udine, ITALY

## Abstract

The science of meditation has grown tremendously in the last two decades. Most studies have focused on evaluating the clinical effectiveness of mindfulness-based interventions, neural and other physiological correlates of meditation, and individual cognitive and emotional aspects of meditation. Far less research has been conducted on more challenging domains to measure, such as group and relational, transpersonal and mystical, and difficult aspects of meditation; anomalous or extraordinary phenomena related to meditation; and post-conventional stages of development associated with meditation. However, these components of meditation may be crucial to people’s psychological and spiritual development, could represent important mediators and/or mechanisms by which meditation confers benefits, and could themselves be important outcomes of meditation practices. In addition, since large numbers of novices are being introduced to meditation, it is helpful to investigate experiences they may encounter that are not well understood. Over the last four years, a task force of meditation researchers and teachers met regularly to develop recommendations for expanding the current meditation research field to include these important yet often neglected topics. These meetings led to a cross-sectional online survey to investigate the prevalence of a wide range of experiences in 1120 meditators. Results show that the majority of respondents report having had many of these anomalous and extraordinary experiences. While some of the topics are potentially controversial, they can be subjected to rigorous scientific investigation. These arenas represent largely uncharted scientific terrain and provide excellent opportunities for both new and experienced researchers. We provide suggestions for future directions, with accompanying online materials to encourage such research.

## Introduction

The field of meditation research has grown exponentially in the past two decades. A total of about 500 peer-reviewed scientific articles on the science of meditation existed in 1990. Today, there are over 4,000 (US National Library of Medicine, pubmed.com). About 25 papers were published per year in the 1990’s, whereas over 400 were published in 2016. This rapid expansion of the field is commendable and has led to a large increase in the knowledge of cognitive, psychological, and neurophysiological changes associated with meditative practices, as well as making important contributions to the current psychotherapeutic armamentarium.

Careful efforts by clinicians, theorists, and researchers to understand meditation have led to a slow but steady shift towards translating meditative practices into clinically relevant interventions, and examining their effects on biological outcomes [1–7]. For example, secularized mindfulness interventions such as MBSR (Mindfulness Based Stress Reduction) and MBCT (Mindfulness Based Cognitive Therapy) have shown therapeutic benefit for managing pain [8–10] improving mental and emotional health [11, 12], and addressing health behaviors such as overeating [13] and substance dependence [14–17]. Large systematic reviews show that contemplative practices infused into 8-week interventions can reduce stress and increase well-being in comparison to active psychotherapeutic control interventions [11, 18, 19]. Mindfulness has also been linked to increased creativity [20], offsetting age-related cognitive decline [21], and improved behavior and attention in youth educational settings [22, 23].

This burgeoning body of research has shed significant light on the effects of meditation practices on basic mechanisms of attention, perception, emotion and cognition [24–26]. A robust new field of contemplative neuroscience has emerged from studies of changes in brain function and structure associated with long-term meditation practice [3, 24] and short-term mindfulness interventions [27–30]. A growing body of literature has been exploring the biological and physiological mechanisms of meditation, including modulation of inflammation, cell-mediated immunity, self-related processing, inhibitory control and protective factors in biological markers of aging [31–33].

While these efforts in meditation science are deeply insightful, there are many outcomes, as well as phenomenological states of conscious and non-conscious processing, that have rarely been examined in the scientific literature to date.

Numerous non-ordinary experiences during or as a result of meditation are described in the texts and teachings of contemplative traditions [34–41]. Some examples of these include: “awakening” or “enlightenment” experiences marked by profound alterations of self-identity, self-narrative and clarity of perception; transcendence of the physical body or out of body experiences; experiences of oneness and interconnectedness; spiritual transmission from teacher to student; dyadic, group, and relational experiences; experiences of non-physical energies (e.g. chi, qi, shakti); mind to mind communication, precognition, veridical perceptions at a distance or extra-sensory perception; past-life recall and reincarnation experiences; synchronicities; experiences of God, deities, and other non-physical entities; and difficult stages of meditation, and periods of disorientation and depersonalization.

With some notable exceptions, most empirical research on meditation does not address these kinds of experiences as components, outcomes, or mechanisms of meditation, in part because these non-ordinary states can be difficult to capture and investigate in laboratory settings. However, anecdotal, survey, and interview data indicate that these aspects of meditation may be more prevalent than is commonly recognized, could represent important mediators or mechanisms by which meditation leads to beneficial cognitive, behavioral, and physiological outcomes [42–44], and could themselves be salient outcomes of meditation practice.

It is generally accepted that meditative practices engender a “witnessing awareness” [35] or meta-awareness of internal and external stimuli that is distinct from ordinary consciousness. Researchers have investigated this and similar constructs as important mediators of the benefits of mindfulness training, including decentering (the ability to observe one’s thoughts and feelings as temporary, objective events in the mind, as opposed to reflections of the self that are necessarily true) [45, 46], metacognitive awareness (in which negative thoughts/feelings are experienced as mental events, rather than as the self) [47], and re-perceiving (being able to disidentify from the contents of consciousness such as thoughts and view moment-by-moment experience with greater clarity and objectivity) [48]. But there are subjectively reported states of awareness that occur during or as a result of meditation that go beyond metacognition.

A small body of research has been conducted into mystical, transcendent, nonlocal or nondual aspects of meditation practice. Tart [49] pioneered scholarly examination of altered states of consciousness produced by meditation practices and their effect on psychological well-being. Goleman [50] surveyed several types of meditation philosophy and practice, which at the time had received scarce attention in psychology or empirical research, noting that most of them focused on changing consciousness and fostering an awakened state or a hypothesized “fifth state of consciousness” [51] characterized by an experience of “pure awareness” in waking life. Transpersonal psychology has explored mystical experiences in depth, relying primarily on theory and qualitative rather than experimental research [52–54]. William James [40], Carl Jung [55, 56], and Abraham Maslow [57] explored these areas as well, although the spiritual or transcendent aspects of their contributions do not often surface in the modern psychotherapeutic or scientific milieu.

More recently, both theoretical descriptions [58] and empirical investigations [59, 60] of subjective experiences of non-duality (a sense of oneness, or a perceived dissolution of the distinction between the observer and the contents of observation) have emerged. These states are thought to occur when the silent background awareness encountered in meditation becomes sufficiently stabilized and integrated with the daily waking experience, so that the habitual reified dualities between subject and object, self and other, in-group and out-group dissipate. These states are hypothesized to lead to a more spacious, compassionate and authentic way of being [61], and appear to have a distinct neurophysiology [60, 62].

A large body of research exists on Transcendental Meditation (TM), a popular mantra-based contemplative practice that is being utilized in secular settings such as schools, hospitals, and business settings. TM is explicitly designed to access and maintain transcendent states (as opposed to other forms of secularized meditation that are designed to reduce stress through meta-cognition, for example) [63]. TM proponents posit that a reduction in mental and physical activity through mantra repetition engenders an experience of “transcendental consciousness,” described as “self-awareness isolated from the processes and objects of experience…characterized by the absence of the very framework (time, space, and body sense) and content (qualities of inner and outer perception) that define waking experiences” (p. 77) [64]. The practice is theorized to normalize various systems in the body, particularly those that engage the sympathetic nervous system and associated hypothalamic-pituitary-adrenal axis in adapting to environmental stressors [65]. Empirical evidence indicates that the transcendent state is neurologically distinct from usual waking, dreaming, or sleep states [66, 67], and it is hypothesized to be responsible for the demonstrated benefits of TM.

There have also been empirical studies of what have been termed “nonlocal” aspects of human consciousness associated with meditation practice. During or as a result of meditation, people report experiences of perceiving information that does not appear limited to the typical five senses or seems to extend across space and time, such as precognition, clairvoyance, and mind-matter interactions (described as “siddhis” in the Hindu yogic traditions) [68]. While controversial, these studies suggest that a history of meditation practice increases the likelihood that laboratory measures of these extended forms of perception will be observed [69–71], indicating that there may be veridical elements of the subjective reports by meditators of timelessness, boundarilessness, and inexplicable perceptual phenomena.

It is possible that these experiences of self-transcendence (defined as the extent to which individuals conceive themselves as integral parts of the universe as a whole [72]), are active ingredients in contemplative practices. Philosophers have proposed that meditation might engender a transformation from a body/ego-based self-identity to a world/universe-centered experience of self not tied to the local body or limited to the self-narrative of the individual practitioner [73]. Some empirical evidence is beginning to emerge supporting this idea. For example, Bormann et al. [59] specifically investigated the spiritual component of a mantram-repetition meditation intervention in veterans, showing that existential spiritual well-being mediated improvement in PTSD symptoms. Another study showed that transcendental meditation decreased anxiety, improved mood, and doubled acute pain tolerance in comparison with secular forms of meditation [74].

Vago and Silbersweig [75] propose a framework for understanding the neurobiological mechanisms of meditation called S-ART—referring to self-awareness, self-regulation, and self-transcendence. Their definition of self-transcendence, “a positive relationship between self and other that transcends self-focused needs and increases prosocial characteristics” (p. 1) is a more psychological definition, but comes close to the forms of transcendence we are proposing might bear further investigation. While there has been an understandable and careful emphasis on secularizing meditation practices for clinical use, it is possible that the mystical, transpersonal, or transcendent aspects of contemplative practices are not only epiphenomena, but could be important outcomes of meditation practice, or mechanisms of action that are in part responsible for positive outcomes such as reduced stress and improved mood.

Between 2013 and 2016, a task force of meditation researchers and teachers met in a series of four three-day working meetings to identify the state of the current literature on this topic and discuss how to broaden the types of constructs being investigated in meditation research. The group ultimately identified several candidate domains that future research can fruitfully pursue. Before moving forward to recommend these domains, a cross-sectional survey was conducted to investigate prevalence and perceived significance of these under-studied experiences among meditation practitioners. The results of this survey were used to guide recommendations for domains of experience most frequently encountered by real-world practitioners.

## Materials and methods

### Participants

Participants were recruited through social media and email distribution, academic list-servs, and online directories of meditation teachers and practitioners. Recruitment was not random, but a wide net was cast to achieve as broad and diverse a sample as possible. In recruitment materials no mention was made of extraordinary, transcendent, or unusual aspects of meditation, to reduce the likelihood of interest in the topic biasing respondents. Instead, participants were told that the survey was designed to assess the prevalence of “personal experiences” during or related to meditation. The only inclusion criterion was having a current or past meditation practice. If participants responded “no” to the survey question “Have you ever practiced meditation?” or if they were below 18 years of age, they were excluded.

### Online survey

Development of the online survey occurred during the third of four 2–3 day working group meetings. We conducted a comprehensive literature review prior to the meetings, to explore whether the domains of meditation research we suspected were understudied had received any substantive research attention. At the first two meetings, we engaged in a collaborative process of mapping the field to determine what domains of meditation research were experiencing growth in funding, interest, and publications as compared to aspects of meditation experiences and outcomes that had received less interest. We engaged in a process of consensus building regarding which categories remained to be pursued with academic rigor. Several categories of under-studied but potentially important domains of meditation experience were identified, and our next step was to determine whether experiences and outcomes associated with those domains were actually experienced by people practicing meditation. Terms were defined, existing measures identified, and items created for constructs without adequate measures to assess the prevalence of such experiences during, after, or related to meditation.

**Mystical and transcendent experiences** were measured with an adapted version of the Revised Mystical Experience Questionnaire (MEQ30). The MEQ30 is a thirty-item questionnaire originally used to measure mystical aspects of psilocybin and other psychedelic compounds’ effects in laboratory studies [76, 77]. The scale has excellent internal consistency for the total score (alpha = .93), and good internal consistency for the four subscales: (1) Mystical, alpha = .93; (2) Positive Mood, alpha = .83; (3) Transcendence of Time and Space, alpha = .81; and (4) Ineffability, alpha = .80. The revised measure asked, “Have you had any of these experiences while meditating?” with respect to 30 experiences such as “Loss of your usual sense of time,” “Sense of awe or awesomeness,” “Experience of amazement and ecstasy,” or “Sense that the experience cannot be described adequately in words.” Response options were 1 = This has never happened to me; 2 = This has happened once; 3 = This has happened 2–5 times; 4 = This has happened many times; or 5 = This almost always happens to me. The maximum mean score for each subscale is 5, and the minimum is 1. Mean scale scores with standard deviations and percentage of total possible were calculated as recommended, and. percentages of respondents endorsing each item are also presented.

#### Extraordinary experiences

To assess the prevalence of and response to other extraordinary experiences, the survey asked about other domains of interest that emerged during the working meetings. Social/Relational items included items such as feeling a strong connection to a meditation teacher, experiencing a sense of collective energy in group meditation, and whether the meditation practice happened in a group, during a retreat, or in a sacred place. Anomalous Physical and Perceptual items included sensations in the body not apparently caused by the physical environment (e.g. heat, cold, tingling), altered sense of vision, hearing, body sensations, smell or taste and breathing, an altered sense of time or space, an altered sense of awareness or identity, increased synchronicities (unlikely coincidences perceived as meaningful), and perception of nonphysical entities (such as a God presence, higher powers, divine beings or angels, demons or negative figures, guides, or other visitors). Experiences related to subjective experiences of extended perception included external physical phenomena (objects moving without apparent physical force), and clairvoyance/telepathy (perceiving information that could not have been known to you by any known physical means, but later turned out to be true)). Difficult States included items such as disturbing feelings of fear, and dread or terror during or after meditation.

Participants were also asked if they communicated any of those experiences to a meditation teacher, and if so 1) whether the teacher was interested or willing to discuss the experiences, 2) how important the teacher thought the experience was, and 3) whether the teacher gave any advice or insight into the experience, and the setting in which the experience happened. To assess perceived importance and valence of the extended perception experiences, because these experiences have been more frequently considered distractions or non-meaningful side-effects of meditation, participants were also asked if those experiences were meaningful to them, how pleasant/unpleasant they found those experiences.

Data were also collected on demographics, current and past religious/spiritual beliefs and practices, meditation experience, and self-reported history of psychological disorders. The survey was administered with the SurveyMonkey platform (http://www.surveymonkey.com) and took approximately 45 minutes to complete. Surveys were administered between November 10, 2014 and February 3, 2015. All research activities were approved by the Institute of Noetic Sciences Institutional Review Board (IRB) and were conducted according to the principles expressed in the Declaration of Helsinki. Written informed consent was obtained from all research participants. The survey instrument and codebook can be found in the S1 and S2 Files supporting information.

### Statistical analysis

Data were retrieved from SurveyMonkey and each entry checked for appropriate values. Since we were primarily interested in prevalence, descriptive statistics were calculated, including means, standard deviations, frequencies, and percentages depending on data type. Data were analyzed in Microsoft Excel 10.0 (Microsoft, Redmond, WA) and STATA/IC 12.1 (Stata Corp, College Station, TX).

## Results

### Demographics

1,856 participants began the survey. 1,793 responded “yes” to having ever practiced meditation and were over 18 years old (those who responded “no” were not asked to continue). Of those, 1,130 participants completed the entire survey. Only data from completers are reported here. Participants were 59% female, and 41% male with an average age of 47 ± 16 (range 18–91). Most participants had some college education (8% high school or equivalent; 20% college/technical school; 33% bachelor’s degree; 24% master’s degree; 15% doctoral degree/professional degree). Meditators from 66 countries around the world participated in the survey. The most represented countries included the United States (57%), Canada (8%), United Kingdom (8%), Australia (4%), India (2%), Portugal (2%), Germany (2%), and New Zealand, Norway, and Mexico (1%), with the remainder (14%) from countries with less than 1% of participants. Twenty-five percent of participants said “Yes” to having ever been formally diagnosed with a psychological disorder, with depression and anxiety being the most prevalent disorders endorsed (Depression- 19%, Anxiety- 14%, Obsessive compulsive- 6%, Eating- 2%, Psychosis- 1%, Impulse control- 1%, Personality- 1%).

### Religion/Spirituality

Participants were asked to indicate their childhood spiritual or religious affiliation. Christianity was the most endorsed affiliation for all participants (73%) with the next highest being None (11%) (see Table 1). Most had a single religious influence growing up, with 7% of participants endorsing multiple religions in childhood. Participants were asked how much this childhood religion or spirituality influenced their upbringing or how much it was part of their family life growing up, with a Likert scale ranging from 0—Not at All to 5- Deeply. The responses were generally evenly distributed. (Not at all- 14%; 1–18%; 2–14%; 3–21%; 5–16%; Deeply- 17%). “*Spiritual but not religious*” was the most endorsed current spiritual or religious affiliation for all participants (Table 1). Religious and spiritual practice was quite important in participants’ current lives, in comparison to in childhood. In response to the question, “How important is your religious or spiritual practice to you now?” 69% of participants rated their practice Very Important, with 17% responding Somewhat Important, 6% A Little Bit Important, and 9% Not Important.

**Table 1:** Childhood and current spiritual or religious affiliations.

	Childhood		Current	
	1 Affiliation (n = 1047)	>1 Affiliation (n = 73)	1 Affiliation (n = 944)	>1 Affiliation (n = 176)
Agnostic	3%	38%	6%	28%
Atheist	4%	36%	3%	18%
Buddhist	1%	14%	11%	62%
Christianity	73%	75%	15%	49%
Hindu	2%	7%	3%	13%
Islamic	1%	1%	1%	2%
Jewish	2%	11%	1%	9%
None	11%	15%	24%	14%
Spiritual but not religious	3%	29%	36%	58%

Values listed are the percent of participants endorsing each affiliation. 7% of respondents reported more than one affiliation in childhood, and 16% reported more than one affiliation currently—for those who reported more than one affiliation, total percentages of respondents reporting each affiliation are reported in the second and fourth column.

### Meditation practice

The average number of years participants engaged in regular (at least once per week) meditation practice was 14.7 ± 13.5 (range 0–75). In the last six months, 71% of participants engaged in “daily” or “more than weekly” meditation practice (Not at all—2%, Less than monthly—4%, Less than weekly, more than monthly—12%, Weekly- 11%, Less than daily, more than weekly—30%, Daily—41%). The most common type of meditation practice was breath-focused followed by open awareness/mindfulness/vipassana (participants could select more than one: Transcendental Meditation—28%, breath-focused—67%, body scan—34%, contemplative prayer—20%, mantra repetition—31%, open awareness/mindfulness—50%, visualization—38%). The most common physical posture was sitting (Sitting- 74%, Laying down- 20%, Walking- 2%, Other- 4%). Most people practiced meditation at least daily or weekly, and more than half of the participants (56%) had completed a multiple-day meditation retreat.

### Mystical experiences

The Revised Mystical Experience Questionnaire (MEQ30) subscale scores are detailed in Table 2. The mean frequencies for all 4 subdomains (Mystical (MYS), Positive Mood (PM), Transcendence of Time and Space (TTS), and Ineffability (IN)) were in the 3.26–3.71 range, indicating frequencies between “2–5 times” (3) and “many times” (4). The Positive Mood domain was most frequently experienced, followed by Ineffability, Transcendence, and Mystical experiences.

**Table 2:** Revised Mystical Experiences Questionnaire (MEQ30) scale scores.

Mystical Experiences	Mean (range 1–5)	Standard Deviation
Mystical	3.26	1.04
Positive Mood	3.71	0.80
Transcendence	3.34	1.03
Ineffability	3.62	1.08
Total	3.39	0.89

**Fig 1:**
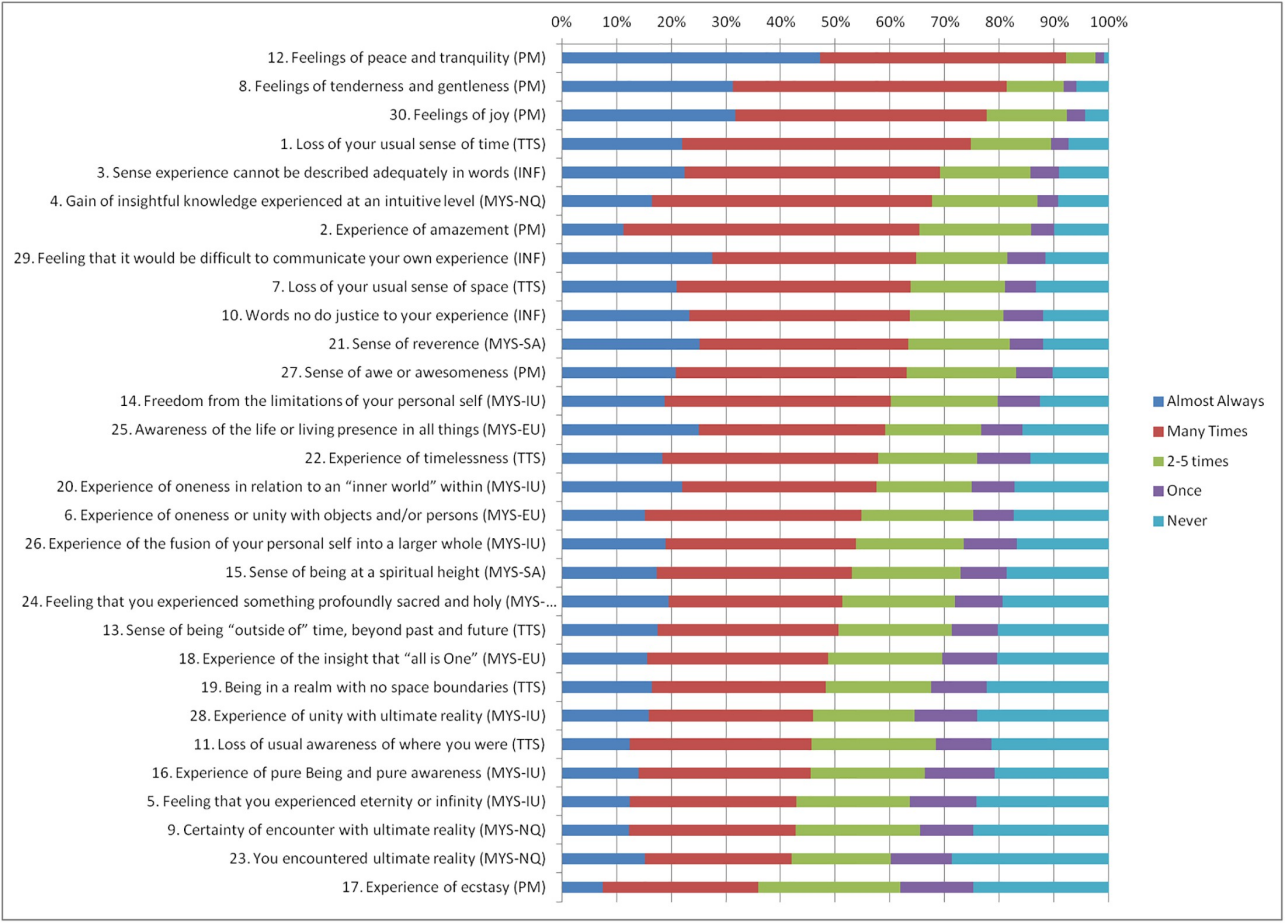
Frequencies of Mystical Experience Questionnaire items. MEQ Subscales: PM = Positive Mood, TTS = Transcendence of Time and Space, INF = Ineffability, MYS = Mystical (Facets of Mystical: MYS-NQ = Noetic Quality, MYS-SA = Sacredness, MYS-IU = Internal Unity, MYS-EU = External Unity). Some items have been truncated due to space. MEQ full items and MEQ subscale scores are available in the supplemental materials.

Frequencies for each item in the Mystical Experiences Questionnaire are shown in Fig 1. Over 40% of respondents reported experiencing all items except one (experience of ecstasy) “many times” or “almost always.”

### Extraordinary experiences

Extraordinary experiences were measured by items newly developed for this study by the working group, arranged into categories including 1) extraordinary physical experiences, 2) spatial-temporal, 3) cognitive-psychological, 4) relational, and 5) extended phenomena. Categories were not combined into subscales, but were used for assessing prevalence of each individual item, and therefore no factor analysis or internal consistency analysis was performed.

The frequencies of these experiences are displayed in Table 3. Altered breathing and sensations in the body that were not apparently caused by the physical environment (such as heat, cold, pressure, tingling or other body sensations) were the most common physical experiences, with 88% and 85% of participants respectively reporting experiencing these at least once, and of those 75% and 73% of people reporting that they had experienced this many times or almost always. Altered sense of time and increased synchronicities were the most common spatio-temporal experiences, with 86% and 82% respectively reporting these and of those, 62% and 65% experiencing them many times or almost always. Altered awareness and aha! moments were the most common cognitive/psychological experiences, with 91% and 89% respectively reporting these experiences, and of those 67% and 62% many times or almost always. Sensing the collective energy of the group was the most common relational experience, reported at least once by 76% of respondents, and many times or always by 47%. Clairvoyance and/or telepathy was the most common extended perception experience, with 56% reporting experiencing this at least once and 30% many times or always The least common, but still quite prevalent, experiences overall were external physical phenomena (objects moving or changing without apparent physical cause) (31%), and disturbing emotions (32%).

**Table 3:** Percentage of participants reporting extraordinary experiences.

			Frequency			
	No	Yes	*One Time*	*2–5 Times*	*Many Times*	*Almost Always*
**Physical**						
Altered breathing	12	88	2	12	42	33
Sensations in body	15	85	4	18	46	17
Altered hearing	20	80	7	23	39	12
Altered vision	28	72	6	22	33	10
Altered body sensations	39	61	5	23	26	7
Altered smell/taste	65	35	5	18	11	1
**Anomalous Spatial-Temporal**						
Altered time	14	86	4	19	44	18
Altered space	40	60	6	21	27	7
Increased synchronicities	18	82	2	15	48	17
Nonphysical entities	40	60	8	20	26	6
**Cognitive/Psychological**						
Altered awareness	9	91	6	18	49	18
Aha! Moments	11	89	4	24	53	9
Altered identity	31	69	8	20	33	9
Disturbing emotions	68	32	9	16	7	0
**Relational**						
Collective energy	24	76	6	23	39	8
Connection with teacher	47	53	8	17	23	5
**Extended Phenomena**						
Clairvoyance/ Telepathy	44	56	6	20	24	6
External Physical Phenomena	69	31	6	16	9	0

Percentage endorsed of each item ordered by most to least common within domain. No = this has never happened. Yes = sum of remaining responses (This has happened once, This has happened 2–5 times, This has happened many times, This almost always happens). Frequency details the percentage of participants who reported each frequency. Full wording for each item is available in online supplemental materials. N = 1,120

### Salience and valence of experiences

To control the length of the survey, and because these experiences have been often pointed to as distractions or non-meaningful side effects, we asked follow-up questions regarding valence and salience of only the two extended perception items (data not shown in table). Participants who had clairvoyant or telepathic experiences (56%) rated the experience as “quite meaningful or important” (mean = 4.01, SD = 1.11; Response Scale: 1 = Not at all, 2 = A little bit, 3 = Somewhat, 4 = Quite a bit, 5 = Very much) and “somewhat pleasant” (mean = 4.10, SD = 1.10; Response scale: 1 = Very Unpleasant, 2 = Somewhat Unpleasant, 3 = Neutral, 4 = Somewhat Pleasant, 5 = Very Pleasant). Participants who had experienced external physical phenomena (31%) rated the experience as “quite meaningful or important” (mean = 4.01, SD = 1.11) and “somewhat pleasant” (mean = 4.07, SD = 1.07).

### Sharing experiences with teachers

Participants were asked “Of the meditation experiences you reported on this survey, which did you mention to a mediation and/or spiritual teacher?” Participants could endorse sharing more than one experience. Six-hundred and one, or just over half of participants reported sharing the following experiences with teachers Mystical/Transcendent n = 414; Unusual Body n = 331; Spatial/Temporal n = 352; Cognitive/psychological n = 426; Relational n = 358; Extended Perception n = 272. The other 519 participants did not report any experiences to a teacher. Teachers were mostly willing to discuss the experiences with the student (11% Not at all, 8% A little bit, 20% Somewhat, 22% Quite a bit, 40% Very much). Many teachers gave the impression that such experiences were important to address and reflect upon (15% Not at all, 10% A little bit, 22% Somewhat, 20% Quite a bit, 33% Very much). Also, many teachers provided insight and/or advice to help integrate and understand the practitioners’ experience(s) (14% Not at all, 12% A little bit, 22% Somewhat, 24% Quite a bit, 28% Very much).

### Context of extraordinary experiences

For each extraordinary experience, participants were asked in what setting the experience occurred. Most extraordinary experiences happened when the meditators were alone (Table 4).

**Table 4:** Percentage of participants reporting mystical and extraordinary experiences by setting.

	Meditating Alone	Meditating at Retreat	Group Meditation	Spontaneous (not during meditation)
Mystical/Transcendent	42	14	21	24
Body	46	13	20	21
Spatial/Temporal	46	15	22	17
Cognitive/Psychological	42	14	19	25
Relational	35	16	29	20
Extended perception	41	10	16	34
Total	42	14	21	23

### Relationship of experiences to length of meditation practice

To explore whether length of meditation experience was related to the frequency with which respondents endorsed items, we conducted Pearson correlations between the self-reported number of months of lifetime meditation practice and reported frequency of mystical and extraordinary experiences. There were small but significant correlations (*p* <.01—*p* < .05, two-tailed, max *r* = .30, max *R*^*2*^ = .09) for all but 7 of the 50 items, excluding “feeling of peace and tranquility,” “feelings of joy,” “an altered sense of your body,” “altered breathing,: “disturbing feelings of fear, dread or terror,” and the importance or valence (pleasantness) of the extended perception items. None of the items were significantly negatively correlated with length of meditation practice. The highest correlations (*p* < .01) between self-reported length of meditation practice and mystical or extraordinary experience items was “clairvoyance or telepathy” (*r* = .30), “feeling that you experienced eternity or infinity” (*r* = .30), and “connection with a teacher or guru who was not physically present, or did not interact with you in any physical way at the time” (*r* = .29).

## Discussion

The results of this survey indicate that mystical and extraordinary experiences are prevalent enough among meditators, and salient enough to those who have them, to warrant further scientific inquiry.

Limitations of this study were that the sample was not randomly selected, and this could limit generalizability to a general sample of meditators. To address this concern, in addition to the masking of the topic of the survey in recruitment materials and recruiting from generalized lists of meditators rather than those known to have a special interest in these domains, we explored whether our sample was different from the general population of modern meditators in their demographics, history of psychiatric disorders, and religious/spiritual background and beliefs. Participants were generally middle-aged, gender-balanced (with slightly more females), and well-educated. Though we are aware of no global population-based surveys of meditation practitioners, these demographics are similar to general survey populations who report meditating [78] and who utilize complementary and alternative medicine in the U.S. [79]. Seventy-five percent of our respondents were from the United States, the UK, and Canada, although participants came from 66 countries around the world. Our participants also represented a broad range of amount of meditation experience and types of practice, which may increase the global generalizability of our findings.

Participants reported slightly higher lifetime prevalence of depression than the general population in the United States (16.6% [80] vs. 19% in this sample) and higher rates of lifetime anxiety disorders (11.8% [81] vs. 14% for anxiety in this sample). There have been few formal studies of anxiety and depression prevalence in a general population of meditators. One large cross-sectional study examined depression and anxiety levels in meditators from Germany and Spain. They found similar levels of depression (19.9%) and anxiety (13.6%) to ours in the German sample, but levels lower than ours in the Spanish sample (depression (6.5%) and anxiety (7.1%)) [82].

There were differences between our respondents and the general population in terms of religious affiliation. Respondents endorsed “spiritual but not religious” (36%) as their current affiliation than any other organized religion, whereas global rates are 16%. Only 15% endorsed Christianity, whereas global surveys list Christianity at 32% [83]. In general, we speculate that our sample was similar enough to a general sample of meditators to make our results likely generalizable, though limited by the lack of population-based random sampling.

Another limitation of our sample is that only 63% of eligible participants who started the survey completed it. This could have led to selection bias. There may also be inherent bias in those who complete a lengthy questionnaire without compensation. A randomly selected population-based survey of meditators would be valuable for future research, as well as replication of this survey. Other limitations include the self-report and retrospective nature of the survey. Future studies could include a prospective study of meditators using daily experience sampling or ecological momentary assessment to capture experiences in real-time.

Since the results of this survey show that experiences associated with the domains identified in our working group are prevalent and frequent, and there is little to no empirical research on them in the literature, the following section provides more robust recommendations and future directions for scientifically pursuing these lines of inquiry.

Readers interested in pursuing any of these domains should refer to the Future of Meditation Research (FOMR) (http://noetic.org/fomr) website for links to papers which provide methods, measures, and protocols for studying these experiences.

### I. Mystical and transcendent experiences in meditation

Experiences that transcend ordinary perception are a common component of religious and spiritual traditions across human history. They can occur spontaneously [84] or can be elicited by a variety of rituals, such as meditation, prayer, fasting, and dance, as well as ingestion of naturally occurring substances (e.g. plants with psychoactive properties) [77, 85–87]. These experiences are not as rare as they might seem. In the general public, 30–50% of people report having had what they would consider a mystical experience [88, 89]. Both historical and modern descriptions of mystical experiences reveal common themes, including feelings of unity and interconnectedness with all people and things, a sense of sacredness, feelings of peace and joy, a sense of transcending normal time and space, ineffability, or an intuitive belief that the experience is a source of objective truth about the nature of reality [89, 90].

Our respondents reported a high frequency of mystical experiences during or related to their meditation practice, the vast majority reporting having them “2–5 times” or “many times” for almost all items. Increased scientific investigations of these experiences may be important to understanding the full range of human potential and well-being.

As reviewed earlier, a common component of many contemplative practices is the recognition of the difference between awareness and the contents of awareness (thoughts, feelings, sensations, etc.). In fact, an “altered sense of awareness, such as awareness going beyond the physical senses, an increased intensity of awareness, or awareness of awareness,” was the most endorsed overall experience (91%) among our participants. Some traditional contemplative theories propose that these experiences spring from awareness recognizing itself [91], or the presence of a background non-conceptual awareness which cognizes without subject-object dichotomy (i.e. “nondual”), and are thought to under certain circumstances be brought into the foreground of experience through the practice of meditation [92]. In this mode, perceptions, emotions, cognitions, and the global states of arousal appear to this awareness as contents, whereas awareness is experienced as a contextual space (like weather patterns appear in the sky). While neuroscience research in this area is still in its very early stages, studies conducted so far indicate that such unitary states are accompanied by increased large scale synchronization and connectivity in the brain [93–95].

Past experiments carried out with split brain patients indicate that the distinction between thoughts and awareness might have a biological basis [96]. Recently, studies have focused on moments when meditators realize that they have lost track of their meditation and are mind wandering, followed by re-orienting of attention on the meditation task [97, 98]. Meta-cognitive or meta-conscious processes are arguably related but different from the awareness responsible for mystical experiences.

In addition, sacred texts in contemplative traditions such as Buddhism and Hinduism claim that meditative practices can result in states of mind that have not been adequately explored or differentiated phenomenologically in the scientific literature. For example, the yoga tradition describes multiple kinds of *samadhi* (states of intense concentration, absorption, calm and equanimity), differentiating between for example, *nirvikalpa samadhi* of pure awareness, and *sahaj samadhi* in which awareness and daily experience both co-arise but are perceived as inseparable, nondual, or coessential [99]. Investigation of these states may offer us new insights about cognition and perception that can only be reached through expanding contemplative science.

Mystical or transcendent aspects of meditation can be challenging to measure, and difficult to predict or produce in a laboratory. With rare exceptions, current research on mystical and transcendent experiences to date rely almost completely on retrospective self-reports using face-valid measures, and are therefore highly open to recall bias and demand characteristics. Future research should focus on better conceptualization and measurement of mystical or transcendent experiences, including objective, implicit, and first, second, and third-person measures. In addition, methods of reliable induction of mystical experiences, and further investigations of those able to produce such experiences at will, may allow for more controlled investigations.

Given the frequency and salience of mystical and transcendent experiences related to meditation practice, we recommend this as a fruitful area for future research. In particular, we suggest conducting studies that 1) investigate the subjective nature and salience of mystical and transcendent experiences, 2) develop improved methods and measures for investigating them, 3) explore the effects of these experiences on health, psychological and prosocial outcomes, 3) examine psychophysiological moderators and mechanisms of such experiences (when, why and how do they happen?), and 4) determine acute and long-term physiological correlates of such experiences. For example, prospective studies of novice meditators could include a measure of mystical or transcendent experiences, examine the predictive value of the occurrence or type of such experiences on outcomes of interest, explore them as potential mechanisms of other psychological or physical changes, or correlate the occurrence and intensity of such experiences with mood data from experience sampling or biomarkers.

### II. Social and relational aspects of meditation

To date, most experimental studies of meditation have focused on cognitive, emotional, and physical correlates of meditation practice within individual subjects. However, meditation has traditionally been taught in a relational manner, from a teacher to a student or in a group of students. There are numerous meditation approaches that encourage meditators to come together to practice, and individuals often find that that meditating in the presence of others can deepen concentration, focus and the overall meditation experience.

Practitioners from a wide variety of spiritual traditions have reported strong psychophysiological responses when they are in the presence of a spiritual teacher who has achieved some level of mastery, particularly when the teacher directs attention or intention toward the practitioner. These reports are common across spiritual traditions, being described most frequently in those that are based in Hinduism and Buddhism. In these traditions, the phenomenon is thought to reflect a “transmission” of a state of consciousness or a form of energy from teacher to student. Recipients also report subjective experiences of receiving such transmissions at a distance, or by listening to a recording or simply looking at a picture of the spiritual teacher. Sensing a collective energy “many times” or “almost always” during meditation practice was endorsed by nearly half of our survey respondents, and three-quarters reported this happening at least once. Connection with a teacher who was not physically present was endorsed “many times” or “almost always” by 28% of respondents, and 45% experienced this at least twice.

Research on social norms and social influence suggests that the mere presence of other people changes the nature of an individual’s experience such that his or her motivations and behavioral choices occur in response to the normative behaviors [100]. Simple examples of this can be found in the social conformity and social facilitation literature [101–103]. The social aspects of meditation practice have just begun to be studied, such as comparing meditation programs taught in groups versus individually [104], and long-term meditation retreats [60, 105]. Interestingly, our sample of meditators reported that mystical/transcendent and extraordinary experiences happened more frequently when meditating alone (35–46% depending on the category of experience) vs. meditating in a group (16–29%) or on retreat (10–16%).

Some questions for future research on the social and relational aspects of meditation include: 1) to what extent does meditating alone vs. meditating in a group of people influence outcomes from biomarkers to mood to behavior? 2) does meditating in a group affect one’s practice positively, negatively, or does it depend on the outcome? 3) do group effects require proximity, or is it enough to know others are meditating at the same time (or asynchronously) in different locations? 4) do group meditation effects depend on personality (such an introversion/extroversion) or other baseline or contextual elements? 5) what is the role of the teacher-student relationship in meditation? 6) are there reliable means of measuring group “energy” or spiritual transmission from teacher to student? 7) what is the impact of meditating with all women or all men, vs. co-ed meditation? and 8) what is the impact of meditating with a significant other? These are intriguing research questions that have only barely been explored. There are also opportunities to study dyadic or group outcomes of meditation practice, such as effects on intimate relationships, work groups, classrooms, or organizations. Multiple simultaneous measures of biomarkers such as heart rate variability or EEG in groups could also be used investigate whether dyadic or group synchrony is detectable, and whether it enhances benefits of meditation.

Furthermore, many goals of meditation practice are specifically oriented toward developing pro-social emotions and behaviors. These include emotions such as love and joy, attitudes such as ethics and altruism, relational skills such as empathy and compassion, virtues such as patience and humility, as well as insights and wisdom about the self and the world [106, 107]. Contemplative science is growing rapidly in studying these prosocial emotions and behaviors related both to meditation practices [108–112] as well as clinical outcomes of compassion and lovingkindness practices [113], but the mechanisms by and extent to which meditation cultivates them are just beginning to be investigated. There remains an enormous opportunity for more work in this promising area.

### III. Physical and perceptual phenomena

Body-based meditation practices are some of the most commonly disseminated techniques in the West. Awareness of the body, particularly awareness of breathing, is a foundational practice across many contemplative traditions. It is not surprising that an “altered sense of breathing” was the body sensation most endorsed by respondents in our survey (88% ever, and 33% almost always).

A large and growing amount of studies have been conducted on physiological correlates of meditation. A variety of research and clinical studies have focused on physical and perceptual outcomes following meditation training, such as changes in autonomic measures [114, 115], tactile and pain perception [116–118], visual and auditory perception [119–122] and even increasing body temperature at will in freezing conditions [123, 124]. In some meditation traditions, practitioners intentionally attempt to control basic physiology, such as respiration rate [125] and heart rate [126].

Physical and perceptual sensations not apparently caused by the physical environment were experienced by the vast majority of our survey respondents, including: heat, cold, pressure, or tingling; seeing lights, visions, or images; lightness or heaviness, floating, out of body experiences, body parts disappearing, or feeling like the body changed in shape or size; hearing buzzing sounds, humming, or voices or music that were not in the physical environment. These are experiences that have rarely been examined in a scientific context, but were endorsed by 60–90% of our respondents. Smelling or tasting things that were not physically there was the least endorsed item, though still reported by 35% of those surveyed.

Some meditation practices focus attention on “energy” flowing through the body. Contemplative traditions each have their own understanding of what this subjectively experienced “energy” is, such as kundalini, chi, or subtle energy, and others describe in detail energy pathways (such as meridians) or nodes (such as chakras) in the body. Many moving meditations such as yoga, qi gong, tai chi, and martial arts are designed for moving or balancing energy in the body, and were at times used to prepare the body for, or used in conjunction with, sitting meditation. These physical phenomena associated with meditation have just barely been addressed by the scientific community, and future studies on these topics could not only help us learn more about the correlates and outcomes of meditation, but also more about the connection between mind and body, and potentially more about what has come to be known as the “biofield” and its role in our well-being [127].

Other outcomes of meditation practice have to do with a visceral sense of greater embodiment, or feeling comfortable, awake, and aware in one’s body. Repeatedly directing attention toward what are typically implicit or automatic body sensations may increase the sense of embodied presence—in other words, experiencing oneself to be fully one’s body in the present moment. Interoceptive awareness (awareness of signals from inside the body) is also an area of increasing interest [128]. While a number of early studies showed that meditators are no better at accurately assessing heart rate than non-meditators [129, 130], other researchers have found increased breath awareness [131], increased heartbeat detection accompanied by increased emotional awareness [132], and increased coherence between subjective assessment of emotion and heart period in trained vipassana meditators [133]. The increasing evidence that humans can become aware of what were previously purely non-conscious processes has profound implications, and provides a large and potentially valuable sphere of scientific inquiry.

Once again, these phenomena certainly provide challenges in terms of measurement and methodology, but so do other areas of inquiry that require ingenuity to operationalize. Future directions for rigorous research on anomalous physical and perceptual phenomena during or as a result of meditation could include 1) qualitative measures to better understand the nature of these experiences; 2) development of quantitative measures to assess subjective experiences of embodiment/physicality, heat, cold, tingling and prickling of the skin, “energy” surges, etc.; 3) objectively measuring physiological correlates of subjective physical, perceptual, or energy experiences; 4) investigations of whether meditative activities can result in functional physical improvements (e.g. strength, balance) or extraordinary capacities for physical performance; or 5) exploring how embodied presence due to meditation practices influence human interactions with virtual or augmented reality (see [134]).

These and other areas of body sensations and perceptual phenomena that occur naturally in meditation provide a rich open field for new research. These lines of inquiry not only provide an opportunity to learn more about the effects of meditation, but also to learn more about mind-body interactions in the context of the special training that meditation practices provide. Just as we learn more about the potential of the human body through Olympic level sports, we might learn more about how the mind and body work together by investigating those with extensive mental training through meditation.

### IV. Spatial/Temporal phenomena

Contemplative practitioners anecdotally report experiencing time and space differently during or as a result of meditation practice. Indeed, an altered sense of time such as regular time seeming shorter or longer than usual, or experiencing awareness in the past or in the future was reported by 86% of our survey participants, with over 60% reporting this “many times” or “almost always.” Over half of our respondents experienced an altered sense of space such as feeling something crackling in the air, sensing something across a distance, or a sense of space being distorted from its usual mode, with over 30% reporting one of these “many times” or “almost always.” Increased synchronicities (meaningful coincidences, or events or information appearing at the same time or place for no apparent reason) were endorsed by 82% of the participants. Indeed, increased synchronicities was the sixth most common experience among all those surveyed (82% having experienced it at least once), even higher than the rate of experiences we might expect from meditation practice, such as altered body sensations.

Recommendations for future research in this domain include: 1) using qualitative research to assess more fully the subjective descriptive nature of meditators’ altered perceptions of time, space, or synchronicities in their lives; 2) using experience sampling, daily assessments, or questionnaires to evaluate the frequency and salience of such experiences; 3) exploring objective physiological correlates of the subjective experience of timelessness [69] or connections with others at a distance (see [135]), or the sense of spaciousness or timelessness (see [136]); 4) assessing the effects of these experiences on identity, decision-making, mood regulation, or other clinical outcomes; and 5) developing methods for reliable induction of these experiences under controlled conditions.

### V. Extended perception

Extended perception refers to perceptions people may have naturally, or develop over the lifespan, that go beyond traditionally understood notions of how information can be perceived. Advanced meditators have demonstrated at least twelve perceptual capacities that scientists once dismissed as impossible [107]. These capacities include, for example, lucid dreaming, lucid nondream sleep, and heightened perceptual speed and sensitivity. What further capacities await recognition?

Over half of the meditators in our sample reported experiencing clairvoyance or telepathy (perceiving information that could not have been known to them by any known physical means, but later turned out to be true) at least once. Not only that, but the majority also found the experience “somewhat pleasant” and “quite meaningful or important.”

Discussions of the relationship between meditation practice and advanced capacities of meditators can be traced in written form back to Patanjali’s Yoga Sutras, published roughly two thousand years ago [36]. Claims such as precognition, clairvoyance, telepathy, and mind-matter interactions are still controversial, although a growing body of literature suggests that some such claims could be supported by data [137–139]. External physical phenomena, or objects moving by a non-physical force, physical objects appearing when they had not been there before, objects falling over, a light going out, psychokinesis (the supposed ability to move objects by mental attention or intention alone), or other physical manifestations that seemed to have no physical cause are also discussed in historical literature. Approximately one-third of the meditators in our sample endorsed having experienced something like this at least once.

People also reported sensing a connection with non-physical entities (defined as nonphysical entities in your awareness, vision, or hearing, such as a God presence, higher powers, divine beings or angels, demons or negative figures, guides, or other visitors) even more often than experiencing a connection with real-life meditation teachers, with 32% reporting this “many times” or “almost always”, and another 52% at least twice.

If new to this literature, scientists encountering these ideas for the first time might argue that if these experiences were prevalent, they would have heard more about them. However, the vast majority of clinicians and researchers do not ask about these experiences in their assessments of meditative practices, and given their controversial nature, modern meditators may be reluctant to share such experiences under non-anonymous conditions. However, many but not all respondents in our survey reported their extraordinary experiences to their meditation teachers. When they did share the experience, they perceived teachers as “somewhat” to “very much” willing to discuss the experience with them, and 75% of teachers gave the impression that they were important to reflect upon, 40% “very much” so.

It is important to note here again that there did not appear to be a substantially higher rate of psychological disorders in this sample than in the general population. While these experiences could be completely illusory, they also could point to aspects of human potential and reality that challenge prevailing paradigms. Western scientists may hesitate to entertain the possibility that one possible explanation for these perceptions of non-local aspects of consciousness are that they are ontologically real. In many meditative traditions, whether they are considered real or not, these experiences are discounted as potentially derailing. Patanjali and others have cautioned that focusing on such experiencing can be seductive, cause egocentricity, or become distractions [140].

At the same time, there are views within some contemplative traditions that such experiences can be utilized with wisdom and compassion by experienced masters, and some highly respected practitioners of contemplative traditions have encouraged more research on such domains. For example, Buddhist monk and collaborator on several neuroscience studies of meditation, Matthieu Ricard was asked at the Mind and Life Institute’s International Symposium on Contemplative Studies in 2012 what he thought would be important for scientists to study next. He responded that reincarnation/past lives and telepathy might be important frontiers to investigate [141], sharing his own personal experience of telepathy with a meditation teacher. Indeed, two of the strongest positive correlations between self-reported length of lifetime meditation practice were with “connection with a teacher or guru who was not physically present” (*r* = .29, r^2^ = .08, *p* < .01) and “clairvoyance or telepathy” (*r* = .30, r^2^ = .09 *p* < .01).

While respecting the concerns of both perspectives, it is possible that the time has arrived to cautiously move beyond earlier assumptions and for investigations to include some of these capacities. Methods currently exist that allow empirical evaluation in the areas described in this paper. Some empirical research already shows that those with a history of meditation practice demonstrate greater “psi” capacities [68, 69, 71, 142–144]. Future directions that intrepid researchers may consider include 1) correlating different types, frequency, and length of meditation practice with a variety of rigorous tests for extraordinary capabilities [145]; 2) testing for extended human capacities such as precognition, clairvoyance, telepathy, or mind-matter interactions under controlled conditions during or just following meditation; 3) utilizing implicit measures (i.e. those that do not require conscious choice but examine physiological or reaction-time measures) to investigate extended human capacities during or related to meditation practice; or 4) including extended human capacities variables or questionnaire items in more traditional studies of meditation, to assess them as predictors, outcomes, or mediators, and 5) studies of people engaging in long-term or high intensity meditation practices who have been reported to exhibit exceptional capacities, virtues, states of consciousness, and postconventional stages of development.

### VI. Other recommendations

#### Difficult experiences in meditation

Meditation is usually considered a low risk intervention and adverse events are relatively rare. While reports of fear and terrors were the least commonly reported type of experience among respondents in our survey, this does not mean that such reports should be ignored. A full 32% of participants in our sample reported feeling disturbing feelings of fear, dread, or terror during or as a result of their meditation practice. A small but growing body of research on adverse effects from meditation practice exists, and there is opportunity to investigate this domain further.

For example, meditation practices have at times been associated with antisocial behavior, restlessness, reduction in emotional stability [146]. Even long term meditators have reported adverse effects [147]. There have been some reports of psychosis and mania triggered by meditation in the scientific literature [148–150] [151] and in lay publications [152]. Further examples include depersonalization [153], and case reports of brain activity correlated with seizures [154, 155]. Generally these findings are consistent with the notion that meditative practices can have powerful effects on mind and body. Changes in self-image and worldview can be signs of psychospiritual progress, but can also be accompanied by significant anxiety. Like other active interventions, significant negative psychological side-effects may occur in a minority of individuals, especially those with a pre-disposition towards mania or psychosis.

Among researchers who are enthusiastic about the benefits of meditation being discovered in contemplative science, there may be hesitance to examine adverse events, negative side effects of meditation, for fear that this will engender fear, restrict research, or lessen enthusiasm for the practice. Most studies do not include any items asking about difficult states or struggles with meditation practice. However, it is possible that difficult and distressing experiences may be involved in one of the major challenges to clinical research on meditation: adherence.

As mentioned earlier, Lindahl and Britton [156] have addressed these questions by collecting data on challenging, difficult, or impairing experiences associated with meditation, the resulting taxonomy of which should aid in encouraging further research. Building and extending this research using a variety of methodologies will only strengthen the field of meditation research. In addition, distressing or difficult states can be viewed as natural aspects of the trajectory of spiritual or contemplative growth, and when properly supported can catalyze positive outcomes [157, 158]. As one American Buddhist teacher, Shinzen Young [159] puts it:

It is certainly the case that almost everyone who gets anywhere with meditation will pass through periods of negative emotion, confusion, disorientation, and heightened sensitivity… for some duration of time, things may get worse before they get better…. This phenomenon, within the Buddhist tradition, is sometimes referred to as “falling into the Pit of the Void.” It entails an authentic and irreversible insight into Emptiness and No Self. … In a sense, it’s Enlightenment’s Evil Twin…In some cases it takes months or even years to fully metabolize, but in my experience the results are almost always highly positive.

Conducting more research on these difficult states and stages should help clinicians help their clients navigate and potentially leverage these experiences.

#### Context

Though not included explicitly in our survey, we recommend that investigation of the role of the environmental context in which meditation practice occurs represents another essentially wide-open field for future researchers. The physical environment, and use of objects, icons, rituals and sacred places have traditionally been thought to enhance meditation practice. There are a potpourri of perceptual cues such as incense, candles, images, music, bells, and the wearing of special clothing, use of sacred foods, or fasting or avoiding certain foods that are routine parts of contemplative traditions and have yet to be investigated scientifically. In some cases, these contextual elements are thought to help “carry” a person into deeper meditative practice, and enhance its benefits.

Environmental cues such as color [160], odor [161], and images [162] have been demonstrated to affect emotion, cognitive processing, and behavior. This may account for the role that environmental cues play in meditation. However, some spiritual lore suggests that buildings, rooms, places, or objects in which many people have engaged in spiritual practices or long periods of meditation feel qualitatively/subjectively different than objects or places that have not been associated with such practices. For example, some talk about the “stillness” or “vibration” of a temple or old church—but objective measures of that subjectively perceived phenomenon are lacking. Only a small amount of research has been conducted on what has been termed “conditioned space [163],” in other words, space that has been purported to be imprinted by intentions alone, and this may warrant further exploration.

In addition, the cultural context, intentions, purpose, and values held by the meditator’s tradition or community (and within the practitioner) likely impact meditative experiences and outcomes. For example, a person who operates from a collectivist cultural orientation [164] might have different experiences of meditative benefit than those who come from more individualistic cultures. Many long term meditation practitioners hold rich worldviews, belief systems and ethical guidelines that inform their motivations for meditative practice and quite possibly the phenomenology of their experiences in meditation. However, the impact of worldview and ethical systems components has not been specifically measured in the bulk of the clinical and neurophysiologic research to date. The novices assayed in meditation research to date hold a broad range of worldviews, often poorly informed by the spiritual and/or religious foundations of the meditative practices in which they are engaging. For better or worse, in clinical settings these meditative practices have by and large been divorced from teachings about ethical guidelines or philosophical understanding about the nature of self and relation of self to world and/or the sacred. There are benefits and drawbacks to this. Secularizing these practices allows for much larger dissemination of them, as well as practices unburdened by dogmas that may or may not be supported by evidence. However, some of the “built-in” ethical protections in traditional settings and teachings have also been stripped away (such as, for example, a meditation student being assigned to clean the temple to learn humility and service while also experiencing transcendent states), and practices run the risk of becoming superficial when decontextualized.

The field of meditation studies is likely to benefit from assessing even in a rudimentary way some of these contextual elements of meditation practice, and how they might impact outcomes. For example, researchers could randomly assign participants to different contextual environments for practice and then collect subjective and objective measurements. One test might include having persons meditate in a room with an object randomly selected as one that is regarded to deepen practice versus a control object. Alternatively, repeated measures designs could also be used in which the same person meditates in various environments, and differences in neurophysiological correlates are measured.

#### Psychological development

One of the most dramatic findings of developmental psychology and neurobiology is that, contrary to previous beliefs, development can continue throughout much of adulthood [165, 166]. There are now more than 100 models of advanced or postconventional [167] stages of adult psychological development [168, 169]. Preliminary maps have been offered over the centuries by contemplatives, but a growing body of empirical research suggests that for moral, cognitive, and many other capacities such as wisdom and self-transcendence, development can continue well into the elder years [170–177]. However, there have been very few studies of the effects of meditation on psychological development, even though accelerating such development may be one of the most important contributions the practice of meditation can make, and one of our contemporary world’s greatest needs.

#### Ethical issues

As the scope of meditation research is broadened, and extraordinary experiences are the increasingly the focus of studies, it will be important to identify and address ethical issues that may arise. Indeed, a barrier to including these experiences and topics in the field of meditation research may have been a concern that too much emphasis on these experiences could encourage people to become distracted from the primary goals of meditation, foster experiences in meditation that could be iatrogenic for patients and clients, or bring to light experiences that clinicians were unequipped to address. However, simply ignoring such experiences does not make them go away, does not preserve the ethical foundations of meditation practice, nor is it an effective clinical approach [178]. Instead, we must create a set of clinical and ethical guidelines for helping clients, students and patients navigate and integrate these experiences to enhance, rather than detract from, their well-being. Educating clinicians and researchers about the potential for these experiences to occur, including questions to screen for distress, depersonalization, or changes in functioning related to meditation practice in assessments, and identifying a clinician with expertise in treating such issues for referrals or consultation are all possible components of an ethical approach.

## Conclusions

The goal of this paper and the accompanying online materials is to share the findings and conclusions reached by the Future of Meditation Research working group. These include the findings of a survey investigating the prevalence of extraordinary meditative experiences and recommendations for expanding future research on meditation. The survey demonstrated that a number of experiences—mystical/transcendent, social/relational, physical/perceptual, and spatial/temporal experiences, and extended human capacities are prevalent and salient to those who experience them, and that meditation teachers are generally willing to discuss them with students.

One theoretical trajectory of psychological and spiritual development through meditation practice could be described in broad strokes as 1) participant comes in with distress or a desire for greater understanding or contentment, 2) through beginning mindfulness practices, the participant learns to stabilize attention, 3) the participant learns to de-center and observe the contents of their awareness or experience rather than being completely fused with their experiences, 4) the participant learns to volitionally make choices about how they wish to approach experiences (e.g. with acceptance, friendly investigation, with contemplation, with simple non-reactive awareness, with compassion), 5) through both subtle and profound insights, realizations, and experiences the participant begins to see themselves and reality as less fixed, is better able to understand context, shifts their sense of identity, and feels a sense of connectedness (less duality) between themselves and others, and 6) through these experiences becomes more compassionate for themselves and others, less reactive, less stressed, and observes improved relationships, less depression and anxiety, and more happiness. The premise of this paper is that in addition to experiences recognized in the contemplative literature as signs of spiritual progress, such as decentering from individual ego-based concerns, the kinds of extra-ordinary experiences we have entertained in this paper may also be important parts of this process.

We propose that these experiences are important to study. They hold the potential not only to shed light on effects of meditation in those who practice it, but may also illuminate new understandings about human potential and the nature of reality. Some of these experiences may be purely subjective or even illusory, but if this is the case, they remain worth investigating to learn more about their functional utility and transformative (or disruptive) potential. In addition, as meditation practice continues to increase in health care settings, it will be important for clinicians to be aware of potentially important, distressing or overwhelming experiences patients may have.

Researchers wishing to explore some of these domains may encounter reluctance, resistance or even ridicule from the scientific and academic community. Many aspects of meditation have been excluded from scientific dialogue to allow contemplative science to mature and be accepted as a field with scientific rigor. A focus on the cognitive and physiological outcomes of meditation, once itself a highly unconventional topic of study, assured that the field of contemplative science would be respected as “hard science,” rather than soft or pseudoscience. The field has understandably de-emphasized what may be essential aspects of meditation by focusing on component parts that are easier to operationalize and more palatable to scientists.

But as shown by our survey results, there are deeper and more mysterious aspects of meditation practice that are worth exploring. Our premise is that these important aspects of meditation are within the bounds of scientific investigation, can and should be studied with scientific rigor, and that their exclusion from scientific dialogue unnecessarily limits our knowledge. Our experience thus far, presenting this research to students and at professional meetings is that 1) researchers are fascinated by these topics, 2) that emerging findings often map on to their personal experiences and observations of students and research participants as well as the spiritual traditions from which many of these practices emerged, and 3) they are gratified to hear that there are intelligent, rigorous, and empirically sound methods to study them. Students and researchers who are interested in investigating these domains of meditation may find it useful to visit the Future of Meditation Research website to find a wealth of references and recommendations, an online course expanding on the topics reviewed in this paper, and a community of researchers who are pursuing these domains of inquiry.

The aim of this paper was to bring attention to some of the more controversial and less studied domains of meditation. We suggest that these aspects of meditation may be crucial to people’s psychological and spiritual development, and rather than being side-effects, could represent important outcomes of meditation practice, or serve as mediators and/or mechanisms by which meditation confers benefits. These arenas represent largely uncharted scientific terrain and provide excellent opportunities for new and experienced researchers. We hope this paper provided a foundation from which future research can expand. We believe it offers preliminary support to Maslow’s [179] provocative claim that “what we call ‘normal’ in psychology is really a psychopathology of the average, so undramatic and so widely spread that we don’t even notice it ordinarily” (p. 16). The intention of this paper is to invite all of us to step into a new paradigm from which to explore one of the greatest of human quests—the understanding, healing, and enhancement of the human mind.

## Supporting information

S1 File Meditation experiences survey. (PDF)

S2 File Meditation experiences survey codebook. (PDF)
