# Supplementary material for: Future directions in meditation research: Recommendations for expanding the field of contemplative science
Source: PLoS One. 2018 Nov 7;13(11):e0205740. doi: 10.1371/journal.pone.0205740 (PMC6221271; doi:10.1371/journal.pone.0205740)
Supplement: S1 File — (PDF) [file pone.0205740.s001.pdf]

Introduction and Consent

Welcome to the Meditation Insight Questionnaire! Thank you for considering telling us about your meditation experience.

This survey asks about experiences you may have had during or related to meditation practice. The majority of questions are multiple choice and address the frequency, quality or intensity of experiences you have had during meditation. The survey takes about 10 to 20 minutes to complete depending on the number of experiences you have had and the depth of further information you provide.

We will use your responses on this survey as the first step in developing an expanded set of concepts to be measured in meditation research. We want to find out how often people report having these experiences as a part of their meditation practice.

We realize that surveys are limited in their capacity to reflect the breadth and depth of experience, and that some questions may be difficult to answer. Whenever possible, please choose the answer that most closely reflects your experience. Also, we recognize that some questions are similar.

Please be aware that completing this survey may cause you discomfort, either due to the time it takes to complete, or the questions themselves.

This survey is completely voluntary and anonymous. We will not collect any information that can be used to identify you unless you choose to give us that information. We will keep the information you provide strictly confidential.

Given the above information, do you agree to participate in this survey?

- ◯ Yes
- ◯ No

Have you ever practiced meditation?

- ◯ Yes
- ◯ No

Experiences During Meditation

Have you had any of these experiences while meditating?

	This has never happened	This has happened once	This has happened 2-5 times	This has happened many times	This almost always happens
Loss of your usual sense of time.					
Experience of amazement.					
Sense that the experience cannot be described adequately in words.					
Gain of insightful knowledge experienced at an intuitive level.					
Feeling that you experienced eternity or infinity.					
Experience of oneness or unity with objects and/or persons perceived in your surroundings.					
Loss of your usual sense of space.					
Feelings of tenderness and gentleness.					
Certainty of encounter with ultimate reality (in the sense of being able to “know” and “see” what is really real) at some time during your experience.					
Feeling that you could not do justice to your experience by describing it in words.					
Loss of usual awareness of where you were.					
Feelings of peace and tranquility.					
Sense of being “outside of” time, beyond past and future.					
Freedom from the limitations of your personal self and feeling a unity or bond with what was felt to be greater than your personal self.					
Sense of being at a spiritual height.					
Experience of pure Being and pure awareness (beyond the world of sense impressions).					
Experience of ecstasy.					
Experience of the insight that “all is One”.					
Being in a realm with no space boundaries.					
Experience of oneness in relation to an “inner world” within.					
Sense of reverence.					
Experience of timelessness.					

	This has never happened	This has happened once	This has happened 2-5 times	This has happened many times	This almost always happens
You are convinced now, as you look back on your experience, that in it you encountered ultimate reality (i.e. that you “knew” and “saw” what was really real).	◯	◯	◯	◯	◯
Feeling that you experienced something profoundly sacred and holy.	◯	◯	◯	◯	◯
Awareness of the life or living presence in all things.	◯	◯	◯	◯	◯
Experience of the fusion of your personal self into a larger whole.	◯	◯	◯	◯	◯
Sense of awe or awesomeness.	◯	◯	◯	◯	◯
Experience of unity with ultimate reality.	◯	◯	◯	◯	◯
Feeling that it would be difficult to communicate your own experience to others who have not had similar experiences.	◯	◯	◯	◯	◯
Feelings of joy.	◯	◯	◯	◯	◯

Experiences During Meditation: Body

During or just after meditating, have you had an experience in your body that was not directly caused by the physical environment (such as heat, cold, pressure, tingling or other body sensations)?

- This has never happened
- This has happened once
- This has happened 2-5 times
- This has happened many times
- This almost always happens

During or just after meditating, have you experienced an altered sense of vision (such as visual images or hallucinations, or seeing lights, geometric patterns, symbols or other visuals that did not seem to be created by the actual physical environment)?

- This has never happened
- This has happened once
- This has happened 2-5 times
- This has happened many times
- This almost always happens

During or just after meditating, have you experienced an altered sense of your body (such as lightness or heaviness, floating, an out of body experience, body parts disappearing, or feeling like your body changed in shape or size)?

- This has never happened
- This has happened once
- This has happened 2-5 times
- This has happened many times
- This almost always happens

During or just after meditating, have you experienced an altered sense of hearing (such as buzzing sounds, humming, hearing voices or music that were not in the physical environment)?

- ◯ This has never happened
- ◯ This has happened once
- ◯ This has happened 2-5 times
- ◯ This has happened many times
- ◯ This almost always happens

During or just after meditating, have you experienced an altered sense of smell or taste (such as smelling or tasting things that were not physically there)?

- ◯ This has never happened
- ◯ This has happened once
- ◯ This has happened 2-5 times
- ◯ This has happened many times
- ◯ This almost always happens

During or just after meditating, have you experienced altered breathing (such as breathing more deeply, more shallowly, or breathing becoming somehow different than usual)?

- ◯ This has never happened
- ◯ This has happened once
- ◯ This has happened 2-5 times
- ◯ This has happened many times
- ◯ This almost always happens

Experiences During Meditation: Spatial/Temporal

During or just after meditating, have you experienced an altered sense of time (such as regular time seeming shorter or longer than usual, or experiencing your awareness in the past or in the future)?

- ○ This has never happened
- ○ This has happened once
- ○ This has happened 2-5 times
- ○ This has happened many times
- ○ This almost always happens

During or just after meditating, have you experienced an altered sense of the space around you (such as feeling something crackling in the air, sensing something across a distance, or another sense of space being distorted from its usual mode)?

- ○ This has never happened
- ○ This has happened once
- ○ This has happened 2-5 times
- ○ This has happened many times
- ○ This almost always happens

During or after meditating, have you experienced increased synchronicities (such as “meaningful coincidences,” or events or information appearing at the same time or for no apparent reason)?

- ○ This has never happened
- ○ This has happened once
- ○ This has happened 2-5 times
- ○ This has happened many times
- ○ This almost always happens

Experiences During Meditation: Cognitive/Psychological

During or just after meditating, have you experienced an Aha! moment, with a new insight or creative solution to a problem that had been vexing you?

- This has never happened
- This has happened once
- This has happened 2-5 times
- This has happened many times
- This almost always happens

During or just after meditating, have you experienced an altered sense of your awareness (such as awareness going beyond your physical senses, an increased intensity of awareness, or awareness of awareness)?

- This has never happened
- This has happened once
- This has happened 2-5 times
- This has happened many times
- This almost always happens

During or just after meditating, have you experienced an altered sense of your identity (such as feeling like someone else, transcending your personal identity, experiencing a new identity or feeling you had no identity)?

- This has never happened
- This has happened once
- This has happened 2-5 times
- This has happened many times
- This almost always happens

During or just after meditating, have you experienced disturbing feelings of fear, dread, or terror?

- This has never happened
- This has happened once
- This has happened 2-5 times
- This has happened many times
- This almost always happens

Experiences During Meditation: Relational

During or just after meditating, have you experienced a sense of collective energy from a group you were physically sitting with, or a group you felt connected to at a distance?

- This has never happened
- This has happened once
- This has happened 2-5 times
- This has happened many times
- This almost always happens

During or just after meditating, have you experienced a connection with a teacher or guru who was not physically present, or did not interact with you in any physical way at the time?

- This has never happened
- This has happened once
- This has happened 2-5 times
- This has happened many times
- This almost always happens

During or just after meditating, have you experienced nonphysical entities in your awareness, vision, or hearing (such as a God presence, higher powers, divine beings or angels, demons or negative figures, guides, or other visitors)?

- This has never happened
- This has happened once
- This has happened 2-5 times
- This has happened many times
- This almost always happens

Experiences During Meditation: Psi

During or just after meditating, have you experienced events in the physical environment, such as things moving when you didn’t think they’d been moved by a physical force, physical objects appearing when they had not been there before, something falling over, a light going out, or other physical manifestations that seemed to have no physical cause?

- ◯ This has never happened
- ◯ This has happened once
- ◯ This has happened 2-5 times
- ◯ This has happened many times
- ◯ This almost always happens

Please briefly describe your experience(s) of events occurring in the physical environment.

In general, how meaningful or important to you was this experience (e.g. did such an experience have an impact on your thinking or feeling, your sense of self, or your worldview)?

- Not at all
- A little bit
- Somewhat
- Quite a bit
- Very much

In general, was this experience pleasant (accompanied by contentment, happiness, joy) or unpleasant (accompanied by anxiety, fear, dread, agitation)?

- Very unpleasant
- Somewhat unpleasant
- Neutral
- Somewhat pleasant
- Very pleasant

Experiences During Meditation: Psi

During or just after meditating, have you experienced clairvoyance or telepathy (in other words you perceived information that could not have been known to you by any known physical means, but later turned out to be true)?

- ◯ This has never happened
- ◯ This has happened once
- ◯ This has happened 2-5 times
- ◯ This has happened many times
- ◯ This almost always happens

Please briefly describe the experience(s) of clairvoyance/telepathy.

In general, how meaningful or important to you was this experience (e.g. did such an experience have an impact on your thinking or feeling, your sense of self, or your worldview)?

- Not at all
- A little bit
- Somewhat
- Quite a bit
- Very much

In general, was the experience pleasant (accompanied by contentment, happiness, joy) or unpleasant (accompanied by anxiety, fear, dread, agitation)?

- Very unpleasant
- Somewhat unpleasant
- Neutral
- Somewhat pleasant
- Very pleasant

Experiences During Meditation: Teacher Dialogue

Of the meditation experiences you reported on in this survey, which did you mention to a meditation and/or spiritual teacher? Please check all the domains that apply.

- [ ] Mystical/Transcendent (e.g., sense of oneness, awe, pure being)
- [ ] Body (e.g., altered vision, hearing, smell, etc.)
- [ ] Spatial/Temporal (e.g. altered sense of time, the space around you)
- [ ] Cognitive/Psychological (e.g., Aha! moments, altered sense of identity, feeling intense emotion)
- [ ] Relational (e.g., sense of connection to a group, teacher, nonphysical entities)
- [ ] Psi (e.g., clairvoyance, telepathy, alteration of physical environment)
- [ ] Did not report any experiences

Please provide anecdotal details for any specific experiences you reported to a meditation or spiritual teacher.

Did the meditation or spiritual teacher seem interested and/or willing to discuss such an experience with you?

- Not at all
- A little bit
- Somewhat
- Quite a bit
- Very much

If the response was mixed (e.g. different experiences elicited different responses), please comment further below:

Overall, did the meditation or spiritual teacher give the impression that such experiences were important for you to address and reflect upon?

- Not at all
- A little bit
- Somewhat
- Quite a bit
- Very much

Did the meditation or spiritual teacher provide you with insight and/or advice to help you integrate and understand your experience(s)?

- Not at all
- A little bit
- Somewhat
- Quite a bit
- Very much

Experiences During Meditation: Setting

The following question asks about the setting or environment in which you may have had certain types of experiences related to meditation.

For the domains below in which you have had an experience related to meditation, please indicate the setting in which it occurred (please check all that apply).

	While meditating on my own	On a meditation retreat	During a group meditation	Spontaneously (not during meditation)
Mystical/Transcendent (e.g., sense of oneness, awe, pure being)	☐	☐	☐	☐
Body (e.g., altered vision, hearing, smell, etc.)	☐	☐	☐	☐
Spatial/Temporal (e.g. altered sense of time, the space around you)	☐	☐	☐	☐
Cognitive/Psychological (e.g., Aha! moments, altered sense of identity, feeling intense emotion)	☐	☐	☐	☐
Relational (e.g., sense of connection to a group, teacher, nonphysical entities)	☐	☐	☐	☐
Psi (e.g., clairvoyance, telepathy, alteration of physical environment)	☐	☐	☐	☐

Demographic Information

Age

Gender

- Male
- Female
- Other

Education Level

- High school or equivalent
- Some college/technical school (2 years)
- Bachelor's degree
- Master's degree
- Doctoral degree/Professional degree (MD, JD, etc.)

What country do you live in?

What is your current spiritual/religious affiliation?

- [ ] Agnostic
- [ ] Atheist
- [ ] Baptist
- [ ] Buddhist
- [ ] Eastern Orthodox
- [ ] Episcopalian
- [ ] Hindu
- [ ] Islamic
- [ ] Jewish
- [ ] LDS (Mormon)
- [ ] Lutheran
- [ ] Methodist
- [ ] Presbyterian
- [ ] Quaker
- [ ] Roman Catholic
- [ ] Seventh Day Adventist
- [ ] Spiritual but not Religious
- [ ] Unitarian/Universalist
- [ ] United Church of Christ
- [ ] New Thought (e.g., Religious Science)
- [ ] Non-denominational Christian
- [ ] None

Other (please specify)

____

What was your childhood spiritual/religious affiliation (i.e., how you were raised)?

- [ ] Agnostic
- [ ] Atheist
- [ ] Baptist
- [ ] Buddhist
- [ ] Eastern Orthodox
- [ ] Episcopalian
- [ ] Hindu
- [ ] Islamic
- [ ] Jewish
- [ ] LDS (Mormon)
- [ ] Lutheran
- [ ] Methodist
- [ ] Presbyterian
- [ ] Quaker
- [ ] Roman Catholic
- [ ] Seventh Day Adventist
- [ ] Spiritual but not religious
- [ ] Unitarian/Universalist
- [ ] United Church of Christ
- [ ] New Thought (e.g., Religious Science)
- [ ] Non-denominational Christian
- [ ] None

Other (please specify)

How much did religion or spirituality influence your upbringing? In other words, how much was religion or spirituality a part of your family life while growing up?

- 0 (Not at all)
- 1
- 2
- 3
- 4
- 5 (Deeply)

How important is your religious or spiritual practice to you now?

- Not important
- A little bit important
- Somewhat important
- Very important

For how many years and/or months (e.g. 4 years and 3 months, or 0 years and 6 months) have you regularly, at least once per week, engaged in a meditation practice?

Years ____________________

Months ____________________

How often have you engaged in a meditation practice over the last 6 months?

- Not at all
- Less than monthly
- Less than weekly, more than monthly
- Weekly
- Less than daily, more than weekly
- Daily

What best describes the kind of meditation you currently or most recently practice (check all that apply)?

- [ ] Transcending
- [ ] Breathing
- [ ] Body Scan
- [ ] Contemplative Prayer
- [ ] Mantra Repetition
- [ ] Open Awareness
- [ ] Visualization

Other (please specify)

Please describe your meditation practice in a few sentences

What is your usual physical posture during meditation?

- ○ Sitting
- ○ Laying down
- ○ Walking
- ○ Other

Have you ever completed a multiple-day meditation retreat?

- ○ Yes
- ○ No

If yes, how many?

Have you ever been formally diagnosed with a psychological disorder?

- ○ Yes
- ○ No
- ○ Decline to state

If you answered yes to the question above, please indicate the relevant domain(s) of disorder below.

- [ ] Anxiety
- [ ] Depression
- [ ] Psychosis
- [ ] Eating
- [ ] Impulse control
- [ ] Personality
- [ ] Obsessive compulsive
- [ ] Post-traumatic stress

Other (please specify)

If you would like to be informed of the results of this survey, please enter your name and email address below.

Name

Email address
