# Supplementary material for: Future directions in meditation research: Recommendations for expanding the field of contemplative science
Source: PLoS One. 2018 Nov 7;13(11):e0205740. doi: 10.1371/journal.pone.0205740 (PMC6221271; doi:10.1371/journal.pone.0205740)
Supplement: S2 File — (PDF) [file pone.0205740.s002.pdf]

Meditation Experiences Questionnaire Codebook
IONS

Q0002- Have you ever practiced meditation?

1=Yes

2=No

The Revised Mystical Experience Questionnaire (MEQ30)

Citation: Barrett, F. S., Johnson, M. W., & Griffiths, R. R. (2015). Validation of the revised Mystical Experience Questionnaire in experimental sessions with psilocybin. *Journal of Psychopharmacology,* 1-9. DOI: 10.1177/0269881115609019.

***Scale responses in survey do not match citation**

MEQ30_1: Loss of your usual sense of time.
MEQ30_2: Experience of amazement.
MEQ30_3: Sense that the experience cannot be described adequately in words.
MEQ30_4: Gain of insightful knowledge experienced at an intuitive level.
MEQ30_5: Feeling that you experienced eternity or infinity.
MEQ30_6: Experience of oneness or unity with objects and/or persons perceived in your surroundings.
MEQ30_7: Loss of your usual sense of space.
MEQ30_8: Feelings of tenderness and gentleness.
MEQ30_9: Certainty of encounter with ultimate reality (in the sense of being able to "know" and "see" what is really real) at some time during your experience.
MEQ30_10: Feeling that you could not do justice to your experience by describing it in words.
MEQ30_11: Loss of usual awareness of where you were.
MEQ30_12: Feelings of peace and tranquility.
MEQ30_13: Sense of being "outside of" time, beyond past and future.
MEQ3014: Freedom from the limitations of your personal self and feeling a unity or bond with what was felt to be greater than your personal self.
MEQ30_15: Sense of being at a spiritual height.
MEQ30_16: Experience of pure Being and pure awareness (beyond the world of sense impressions).
MEQ30_17: Experience of ecstasy.
MEQ30_18: Experience of the insight that "all is One".
MEQ30_19: Being in a realm with no space boundaries.
MEQ30_20: Experience of oneness in relation to an "inner world" within.
MEQ30_21: Sense of reverence.
MEQ30_22: Experience of timelessness.
MEQ30_23: You are convinced now, as you look back on your experience, that in it you encountered ultimate reality (i.e. that you "knew" and "saw" what was really real).
MEQ30_24: Feeling that you experienced something profoundly sacred and holy.
MEQ30_25: Awareness of the life or living presence in all things.
MEQ30_26: Experience of the fusion of your personal self into a larger whole.
MEQ30_27: Sense of awe or awesomeness.
MEQ30_28: Experience of unity with ultimate reality.
MEQ30_29: Feeling that it would be difficult to communicate your own experience to others who have not had similar experiences.
MEQ30_30: Feelings of joy.

1=This has never happened to me.
2=This has happened once.

3=This has happened 2-5 times.
4=This has happened many times
5=This almost always happens to me.

Mystical:
```
COMPUTE
SC_MEQ30_Mystical=MEAN(MEQ30_4,MEQ30_5,MEQ30_6,MEQ30_14,MEQ30_15,MEQ30_16,MEQ30_18,
MEQ30_20,
    MEQ30_21,MEQ30_23,MEQ30_24,MEQ30_25,MEQ30_26,MEQ30_28).
EXECUTE.
```

ALPHA= .949

Positive Mood:
```
COMPUTE
SC_MEQ30_PositiveMood=MEAN(MEQ30_2,MEQ30_8,MEQ30_12,MEQ30_17,MEQ30_27,MEQ30_30).
EXECUTE.
```

ALPHA= .946

Transcendence:
```
COMPUTE
SC_MEQ30_Transcendence=MEAN(MEQ30_1,MEQ30_7,MEQ30_11,MEQ30_13,MEQ30_19,MEQ30_22).
EXECUTE.
```

ALPHA= .888

Ineffability:
```
COMPUTE SC_MEQ30_Ineffability=MEAN(MEQ30_3,MEQ30_10,MEQ30_29).
EXECUTE.
```

ALPHA= .856

Total:
```
COMPUTE
SC_MEQ30_Total=MEAN(MEQ30_1,MEQ30_2,MEQ30_3,MEQ30_4,MEQ30_5,MEQ30_6,MEQ30_7,MEQ30
_8,MEQ30_9,MEQ30_10,MEQ30_11,MEQ30_12,MEQ30_13,MEQ30_14,MEQ30_15,MEQ30_16,MEQ30_17,
MEQ30_19,MEQ30_20,MEQ30_21,MEQ30_22,MEQ30_23,MEQ30_24,MEQ30_25,MEQ30_26,MEQ30_27,M
EQ30_28,MEQ30_29,MEQ30_30).
EXECUTE.
```

ALPHA= .965

If you report scores, we recommend that you report them as the percentage of the maximum possible score.
Let me know if I can help any further.

Experiences During Meditation
TOTAL SCORE

Q4 through Q20
ALPHA= .892

```
COMPUTE
SC_Exp_Meditation_Total=MEAN(q4,q5,q6,q7,q8,q9,q10,q11,q12,q13,q14,q15,q16,q17,q18,
q19,q20).
EXECUTE.
```

Experiences During Meditation: Body

q4: During or just after meditating, have you had an experience in your body that was not directly caused by the physical environment (such as heat, cold, pressure, tingling or other body sensations)?
q5: During or just after meditating, have you experienced an altered sense of vision (such as visual images or hallucinations, or seeing lights, geometric patterns, symbols or other visuals that did not seem to be created by the actual physical environment)?
q6: During or just after meditating, have you experienced an altered sense of your body (such as lightness or heaviness, floating, an out of body experience, body parts disappearing, or feeling like your body changed in shape or size)?
q7: During or just after meditating, have you experienced an altered sense of hearing (such as buzzing sounds, humming, hearing voices or music that were not in the physical environment)?
q8: During or just after meditating, have you experienced an altered sense of smell or taste (such as smelling or tasting things that were not physically there)?
q9: During or just after meditating, have you experienced altered breathing (such as breathing more deeply, more shallowly, or breathing becoming somehow different than usual)?

1=This has never happened.
2=This has happened once.
3=This has happened 2-5 times.
4=This has happened many times.
5=This almost always happens.

```
COMPUTE SC_Exp_Meditation_Body=MEAN(q4,q5,q6,q7,q8,q9).
EXECUTE.
```

ALPHA= .776

Experience During Meditation: Spatial/Temporal

q10: During or just after meditating, have you experienced an altered sense of time (such as regular time seeming shorter or longer than usual, or experiencing your awareness in the past or in the future)?

q11: During or just after meditating, have you experienced an altered sense of the space around you (such as feeling something crackling in the air, sensing something across a distance, or another sense of space being distorted from its usual mode)?

q12: During or after meditating, have you experienced increased synchronicities (such as "meaningful coincidences," or events or information appearing at the same time or for no apparent reason)?

1=This has never happened.
2=This has happened once.
3=This has happened 2-5 times.
4=This has happened many times.
5=This almost always happens.

```
COMPUTE SC_Exp_Meditation_Spatial_Temp=MEAN(q10,q11,q12).
EXECUTE.
```

ALPHA= .668

Experiences During Meditation: Cognitive/Psychological

q13: During or just after meditating, have you experienced an Aha! moment, with a new insight or creative solution to a problem that had been vexing you?

q14: During or just after meditating, have you experienced an altered sense of your awareness (such as awareness going beyond your physical senses, an increased intensity of awareness, or awareness of awareness)?

q15: During or just after meditating, have you experienced an altered sense of your identity (such as feeling like someone else, transcending your personal identity, experiencing a new identity or feeling you had no identity)?

q16: During or just after meditating, have you experienced disturbing feelings of fear, dread, or terror?

1=This has never happened.
2=This has happened once.
3=This has happened 2-5 times.
4=This has happened many times.
5=This almost always happens.

```
COMPUTE SC_Exp_Meditation_Cognitive=MEAN(q13,q14,q15,q16).
EXECUTE.
```

ALPHA= .640

Experiences During Meditation: Relational

q17: During or just after meditating, have you experienced a sense of collective energy from a group you were physically sitting with, or a group you felt connected to at a distance?

q18: During or just after meditating, have you experienced a connection with a teacher or guru who was not physically present, or did not interact with you in any physical way at the time?

q19: During or just after meditating, have you experienced nonphysical entities in your awareness, vision, or hearing (such as a God presence, higher powers, divine beings or angels, demons or negative figures, guides, or other visitors)?

1=This has never happened.
2=This has happened once.
3=This has happened 2-5 times.
4=This has happened many times.
5=This almost always happens.

```
COMPUTE SC_Exp_Meditation_Relational=MEAN(q17,q18,q19).
EXECUTE.
```

ALPHA= .697

Experiences During Meditation: Psi

q20: During or just after meditating, have you experienced events in the physical environment, such as things moving when you didn't think they'd been moved by a physical force, physical objects appearing when they had not been there before, something falling over, a light going out, or other physical manifestations that seemed to have no physical cause.

1=This has never happened.
2=This has happened once.
3=This has happened 2-5 times.
4=This has happened many times.
5=This almost always happens.

Physical Environment

q21: Please briefly describe your experience(s) of events occurring in the physical environment.

Open comment field/Free response.

q22: In general, how meaningful or important to you was this experience (e.g. did such an experience have an impact on your thinking or feeling, your sense of self, or your worldview)?

1=Not at all
2=A little bit
3=Somewhat
4=Quite a bit
5=Very much

q23: In general, was this experience pleasant (accompanied by contentment, happiness, joy) or unpleasant (accompanied by anxiety, fear, dread, agitation)?

1=Very unpleasant
2=Somewhat unpleasant
3=Neutral
4=Somewhat pleasant
5=Very pleasant

Psi

q24: During or just after meditating, have you experienced clairvoyance or telepathy (in other words you perceived information that could not have been known to you by any known physical means, but later turned out to be true)?

1=This has never happened.
2=This has happened once.
3=This has happened 2-5 times.
4=This has happened many times.
5=This almost always happens.

q25: Please briefly describe the experience(s) of clairvoyance/telepathy.

Open comment field/Free response.

q26: In general, how meaningful or important to you was this experience (e.g. did such an experience have an impact on your thinking or feeling, your sense of self, or your worldview)?

1= Not at all
2= A little bit
3= Somewhat
4= Quite a bit
5= Very much

q27: In general, was the experience pleasant (accompanied by contentment, happiness, joy) or unpleasant (accompanied by anxiety, fear, dread, agitation)?

1= very unpleasant
2= somewhat pleasant
3= neutral
4= somewhat pleasant
5= very pleasant

Teacher Dialogue

q28: Of the meditation experiences you reported on this survey, which did you mention to a mediation and/or spiritual teacher? Please check all the domains that apply.

a) Mystical/Transcendent (e.g. sense of oneness, awe, pure being)
   1=yes
b) Body (e.g. altered vision, hearing, smell, etc.)
   1=yes
c) Spatial/Temporal (e.g. altered sense of time, the space around you)
   1=yes
d) Cognitive/Psychological (e.g. Aha! Moments, altered sense of identity, feeling intense emotion)
   1=yes
e) Relational (e.g. sense of connection to a group, teacher nonphysical entities)
   1=yes
f) Psi (e.g. clairvoyance, alteration of physical environment)
   1=yes
g) Did not report any experience
   1=yes

q29: Please provide anecdotal details for any specific experiences you reported to a meditation or spiritual teacher.

Open comment field/Free response.

q30: Did the meditation or spiritual teacher seem interested and/or willing to discuss such an experience with you?

1= Not at all
2= A little bit
3= Somewhat
4= Quite a bit
5= Very much

q30_other: If the response was mixed (e.g. different experiences elicited different responses), please comment further below:

Open comment field/Free response.

q31: Overall, did the meditation or spiritual teacher give the impression that such experiences were important for you to address and reflect upon?

1= Not at all
2= A little bit
3= Somewhat
4= Quite a bit

5= Very much

Q32: Did the meditation or spiritual teacher provide you with insight and/or advice to help you integrate and understand your experience(s)?

1= Not at all
2= A little bit
3= Somewhat
4= Quite a bit
5= Very much

Experiences During Meditation: Setting

q33: For the domains below in which you have had an experience related to meditation, please indicate the setting in which it occurred (Please check all that apply).

a) Mystical/Transcendent (e.g., sense of oneness, awe, pure being)
b) Body (e.g., altered vision, hearing, smell, etc.)
c) Spatial/Temporal (e.g. altered sense of time, the space around you)
d) Cognitive/Psychological (e.g., Aha! moments, altered sense of identity, feeling intense emotion)
e) Relational (e.g., sense of connection to a group, teacher, nonphysical entities)
f) Psi (e.g., clairvoyance, telepathy, alteration of physical environment)

1=While meditating on my own
2=On a meditation retreat
3= During a group meditation
4= Spontaneously (not during meditation)

Demographics

age

gender

1=male
2=female
3=other

educ: Education level

1= High school or equivalent
2= Some college/technical school (2 years)
3=Bachelor's degree
4=Master's degree
5= Doctoral degree/Professional degree (MD, JD, etc.)

County: What country do you live in?

Open comment field/Free response.

q34: What is your current spiritual/religious affiliation?

a. Agnostic
b. Atheist
c. Baptist
d. Buddhist
e. Easter orthodox
f. Episcopalian
g. Hindu
h. Islamic
i. Jewish
j. LDS (Mormon)
k. Lutheran
l. Methodist
m. Presbyterian
n. Quaker
o. Roman Catholic
p. Seventh Day Adventist
q. Spiritual but not religious
r. Unitarian/Universalist
s. United Church of Christ
t. New Thought (e.g. Religious Science)
u. Non-denominational Christian
v. None
w. Other

Q34c+q34e+q34f+q34j+q34k+ q34l+ q34m+ q34n+ q34o+ q34p+ q34r+ q34s+ q34t+ q34u

q35: What is your childhood spiritual/religious affiliation?

a. Agnostic
b. Atheist

c. Baptist
d. Buddhist
e. Easter orthodox
f. Episcopalian
g. Hindu
h. Islamic
i. Jewish
j. LDS (Mormon)
k. Lutheran
l. Methodist
m. Presbyterian
n. Quaker
o. Roman Catholic
p. Seventh Day Adventist
q. Spiritual but not religious
r. Unitarian/Universalist
s. United Church of Christ
t. New Thought (e.g. Religious Science)
u. Non-denominational Christian
v. None
w. Other

q36: How much did religion or spirituality influence your upbringing? In other words, how much was religion or spirituality a part of your family life while growing up?

0=0 (not at all)
1=1
2=2
3=3
4=4
5=5 (deeply)

q37: How important is your religious or spiritual practice to you now?

1=Not important
2=A little bit important
3= Somewhat important
4= Very important

q.38: For how many years and/or months (e.g. 4 years and 3 months, or 1 years and 6 months) have you regularly, at least once per week, engaged in a meditation practice?

a. Years
b. Months

q39: How often have you engaged in a meditation practice over the last 6 months?

1= Not at all

2= Less than monthly
3= Less than weekly, more than monthly
4= Weekly
5= Less than daily, more than weekly
6= Daily

q40: What best describes the kind of meditation you currently or most recently practice (check all that apply)?

a. Transcending
b. Breathing
c. Body Scan
d. Contemplative Prayer
e. Mantra Repetition
f. Open Awareness
g. Visualization
h. Other

q41: Please describe your meditation practice in a few sentences.

q42: What is your usual physical posture during meditation?

1=sitting
2=laying down
3=walking
4=other

q43: Hove you ever completed a multiple-day meditation retreat?
1=yes
2=no

q43a: If yes, how many?

q44: Have you ever been formally diagnosed with a psychological disorder?

1=yes
2=no

a. Anxiety
b. Depression
c. Psychosis
d. Eating
e. Impulse control
f. Personality
g. Obsessive compulsive
h. Post-traumatic stress
i. Other

Name: Name

Email: Email address
